# Investigating the Association between Flowering Time and Defense in the *Arabidopsis thaliana-Fusarium oxysporum* Interaction

**DOI:** 10.1371/journal.pone.0127699

**Published:** 2015-06-02

**Authors:** Rebecca Lyons, Anca Rusu, Jiri Stiller, Jonathan Powell, John M. Manners, Kemal Kazan

**Affiliations:** 1 CSIRO Agriculture Flagship, Queensland Bioscience Precinct, Brisbane, QLD, 4067, Australia; 2 CSIRO Agriculture Flagship, Black Mountain Laboratories, Canberra, ACT, 2601, Australia; 3 Queensland Alliance for Agriculture & Food Innovation (QAAFI), The University of Queensland, St Lucia, Brisbane, Queensland 4067, Australia; National Taiwan University, TAIWAN

## Abstract

Plants respond to pathogens either by investing more resources into immunity which is costly to development, or by accelerating reproductive processes such as flowering time to ensure reproduction occurs before the plant succumbs to disease. In this study we explored the link between flowering time and pathogen defense using the interaction between *Arabidopsis thaliana* and the root infecting fungal pathogen *Fusarium oxysporum*. We report that *F*. *oxysporum* infection accelerates flowering time and regulates transcription of a number of floral integrator genes, including FLOWERING LOCUS C (FLC), FLOWERING LOCUS T (FT) and GIGANTEA (GI). Furthermore, we observed a positive correlation between late flowering and resistance to *F*. *oxysporum* in *A*. *thaliana* natural ecotypes. Late-flowering *gi* and autonomous pathway mutants also exhibited enhanced resistance to *F*. *oxysporum*, supporting the association between flowering time and defense. However, epistasis analysis showed that accelerating flowering time by deletion of FLC in *fve-3* or *fpa-7* mutants did not alter disease resistance, suggesting that the effect of autonomous pathway on disease resistance occurs independently from flowering time. Indeed, RNA-seq analyses suggest that *fve-3* mediated resistance to *F*. *oxysporum* is most likely a result of altered defense-associated gene transcription. Together, our results indicate that the association between flowering time and pathogen defense is complex and can involve both pleiotropic and direct effects.

## Introduction

Plants are frequently attacked by pathogens and deploy chemical and structural barriers to defend themselves, diverting plant resources away from growth and development [1]. To ensure the plant survives to reproduce, the timing of the transition to flowering and the amplitude of the immune response are tightly regulated. Plants often respond to biotic stress by altering flowering time. For instance, susceptible Arabidopsis plants infected by bacterial and oomycete pathogens flower earlier than uninoculated plants [2] while herbivory by the African cotton leafworm *Spodoptera littoralis* delays flowering in *Brassica rapa* [3]. Flowering time was also recently shown to be dependent on soil properties and soil microbiota in a wild relative of Arabidopsis [4].

Defense phytohormones and associated signaling pathways have been shown to alter the transition to flowering. For instance, salicylic acid (SA)-deficient Arabidopsis mutants and transgenic plants such as *sid2* and *eds5* and *NahG* show delayed flowering [5], while the SA regulators WIN3 and NPR1 repress flowering [6]. Regulators of SA-mediated defenses such as SUMO E3 ligase SIZ1, PLANT U-BOX 13 (PUB13) and MYB30 also regulate flowering time [7–9]. The jasmonate (JA) receptor mutant *coi1* is early flowering [10]; plants that are touched repeatedly show a JA-dependent delay in flowering [11] and a subgroup of bHLH transcription factors that negatively regulate JA-mediated defense responses promote flowering [10]. Ethylene (ET)-insensitive mutants are late-flowering [12] and the histone deacetylases HDA6 and HDA19 that are required for JA and ET- mediated defense responses are thought to promote the transition to flowering [13–15].

In *Arabidopsis thaliana*, the transition from vegetative to reproductive growth is a complex trait regulated by an elaborate network of genetic pathways, including the vernalization, photoperiod, thermosensory, autonomous and gibberellin (GA) pathways [16]. Recent evidence shows defense-associated roles for Arabidopsis genes originally identified as regulators of flowering. For instance, FPA and FLD, members of the autonomous pathway, promote susceptibility to the bacterial pathogen *Pseudomonas syringae* [17–19] while the floral meristem identity gene LEAFY represses key regulators of basal immunity [20]. More recently, the phytohormones GA and brassinosteroids that regulate flowering time have also been implicated in defense regulation [21].

*Fusarium oxysporum* is a ubiquitous soil-borne root infecting fungal pathogen that causes vascular wilt diseases of several plant species including *A*. *thaliana* [22]. In the *F*. *oxysporum* – *A*. *thaliana* interaction, resistance is thought to be inherited as a quantitative trait [23–25]. *F*. *oxysporum* infects the plant via lateral root initials and enters the xylem where it travels to the shoots [26, 27]. During the early stages of infection, *F*. *oxysporum* acts as biotroph, gaining nutrition from living tissue. As infection progresses, *F*. *oxysporum* switches from a biotrophic to necrotrophic lifestyle, in which fungal nutrition is gained from necrotic host tissue. In this stage of infection, the host plant exhibits leaf chlorosis, necrosis and senescence. *F*. *oxysporum* produces bioactive JAs, [28], which presumably promote host senescence to accelerate the transition from the biotrophic to necrotrophic phase of infection.

Several late-flowering Arabidopsis mutants including *phytochrome and flowering time1* (*pft1*), *mediator 8 (med8)*, *myc2* and *auxin response factor 2* (*arf2*) show enhanced resistance to *F*. *oxysporum* [29, 30, 31], suggesting interplay between flowering time and defense in the *F*. *oxysporum-A*. *thaliana* interaction. Quantitative trait loci conferring resistance to *Verticillium* spp, a hemibiotrophic fungal pathogen causing vascular wilt disease, have not yet been cloned, but map to regions containing flowering-time genes in *A*. *thaliana* [32, 33].

In this study, we investigated the relationship between flowering time and defense in the *F*. *oxysporum – A*. *thaliana* interaction. Firstly, we investigated the effect of *F*. *oxysporum* infection on the transition to flowering in the host. Secondly, we investigated the response of natural *A*. *thaliana* ecotypes and *A*. *thaliana* flowering-time mutants to *F*. *oxysporum* infection and found a correlation between late flowering time and *F*. *oxysporum* resistance. Interestingly, the observed association was independent from vernalization and the flowering repressor FLC in late-flowering mutants including *fve-3*, leading us to further investigate the mechanism underlying enhanced resistance in *fve-3* using RNA-seq analyses. Finally, we identified *F*. *oxysporum*-responsive flowering-time genes using RNA-seq analyses and found that the photoperiodic pathway regulator GIGANTEA promotes susceptibility to *F*. *oxysporum*.

## Materials and Methods

### Plant material and growth conditions

Eighty-three *A*. *thaliana* ecotypes (stock CS22660) were acquired from the Arabidopsis Biological Resource Centre (ABRC). Mutants are in a Col-0 background unless otherwise specified. The following mutants have been previously described: *fpa-8*, *fpa-7*, *fy-2*, *sr45-1*, *flk-1*, *fld-3 and ref-6-3* [34]; *fve-3*, *fve-2* (Ler) and *fy-1* [35]), *fld-2* (Ler) [36], *fve-3/flc-3*, *fpa-7/flc-3* and *flc-3* [37], *fve-2* (Ler), *fld-2* (Ler), *fy-1* (Ler) and ColFRI^SF2^ [38], *vin3-4* (ColFRI^SF2^) [39], *gi-1* (Col-1) and *gi-2* (Col-1) [40]. To compare vernalized and non-vernalized plants, seeds for vernalization were placed on damp soil for 6 weeks in the dark at 4°C. Two days before end of the vernalization period, the non-vernalized control seeds were stratified for 2 days at 4°C. All seedlings were then grown concurrently. Plants were grown under short day conditions (8 h photoperiod, 21°C, photosynthetically active radiation (PAR) 70–85μmol m^-2^ s^-1^ and relative humidity %RH ≥80%).

### Flowering time measurement

Flowering time was measured as the number of days taken from germination until emergence of a 1cm bolt in healthy, uninoculated plants. At least two plants were assessed per line. Plants that had not flowered by the termination of the experiment were allocated a ‘flowering time’ equal to the number of days for which the experiment had run. This was either 80 or 200.

### *F*. *oxysporum* inoculation and disease assessment

The *F*. *oxysporum* isolate used in this study was strain Fo5176 obtained from Dr Roger Shivas, Queensland Plant Pathology Herbarium, Brisbane, Australia. Inoculations were performed as described previously [41]. Briefly, roots of 4-week-old plants which had been grown under short day conditions (8 h photoperiod) at 21°C were dipped in a *F*. *oxysporum* suspension containing 1 × 10^6^ spores ml^−1^, replanted and placed under long day growth conditions (16 h photoperiod) at 28°C (PAR = 72-80umol m^-2^ s^-1^ and relative humidity %RH ≥90%). Disease was measured by visually assessing symptom development on the leaves at 14 dpi either using a scale of 0–5 with 0 being asymptomatic and 5 being dead as described previously [42] or by percentage of diseased leaves [43]. Readings were then normalized back to Col-0 for each tray to account for tray-to-tray variability. Three to 40 plants were assessed per line. Each tray contained the susceptible ecotype Ty-0 as a positive control.

### Statistical analyses

To assess the statistical significance of a linear correlation between flowering time or latitude and disease score, data were subjected to Pearson's product-moment correlation test using ‘R’ version 3.0.3. A Student’s t–test was used to identify *A*. *thaliana* ecotypes or mutants which were significantly more resistant or susceptible compared to Col-0 or Ler-0 (*fve-2* and *fld-2*) using Excel.

### RNA-seq analysis

Leaves and roots from *fve-3* and Col-0 plants inoculated with either water (mock treatment) or *F*. *oxysporum* were harvested and total RNA was extracted and DNAse treated using the RNeasy mini kit (Qiagen) according to the manufacturer’s instructions. RNA integrity was confirmed using the Agilent 2100 bioanalyser Plant Nano system (Agilent Biotechnologies). Library preparation and sequencing were performed by the Australian Genome Research Facility (AGRF). Messenger RNA was selected using Poly-A tail selection prior to preparation of 50bp single end read libraries. Sequencing was performed on an Illumina HiSeq 2000 system generating approximately from 6.5 to 16 million raw RNA-seq reads per sample.

Differential expression analysis was performed using the Tuxedo analysis suite [44]. Briefly, Bowtie2 along with Tophat were used to align generated reads to the TAIR10 *A*. *thaliana* reference genome. After expressed transfrags were assembled, Cufflinks was used to quantify gene abundance and transcriptome assemblies were then merged using Cuffmerge. Cuffdiff was then performed to identify genes differentially expressed by *F*. *oxysporum* in Col-0 or genes differentially expressed in *fve-3* relative to Col-0. Statistical analysis was performed within the Cufflinks analysis with false discovery rate and correction for multiple comparisons applied using standard run parameters. Genes considered differentially expressed showed a statistically significant difference in expression values (*P*<0.05). 0.6–2.2% of reads did not map to the *A*. *thaliana* genome. Sequence data are available from NCBI under Sequence Read Archive (SRA) accession SRP052276.

Reads that did not align to annotated transcripts were omitted from the analysis. For reads that mapped to two transcripts, the least significantly aligned transcript(s) were omitted. To determine the functionality of genes differentially expressed in *fve-3* plants, genome ontology (GO) enrichment analysis was performed using http://bioinfo.cau.edu.cn/agriGO/ [45].

## Results

### *F*. *oxysporum* infection accelerates the transition to flowering

**Fig 1:**
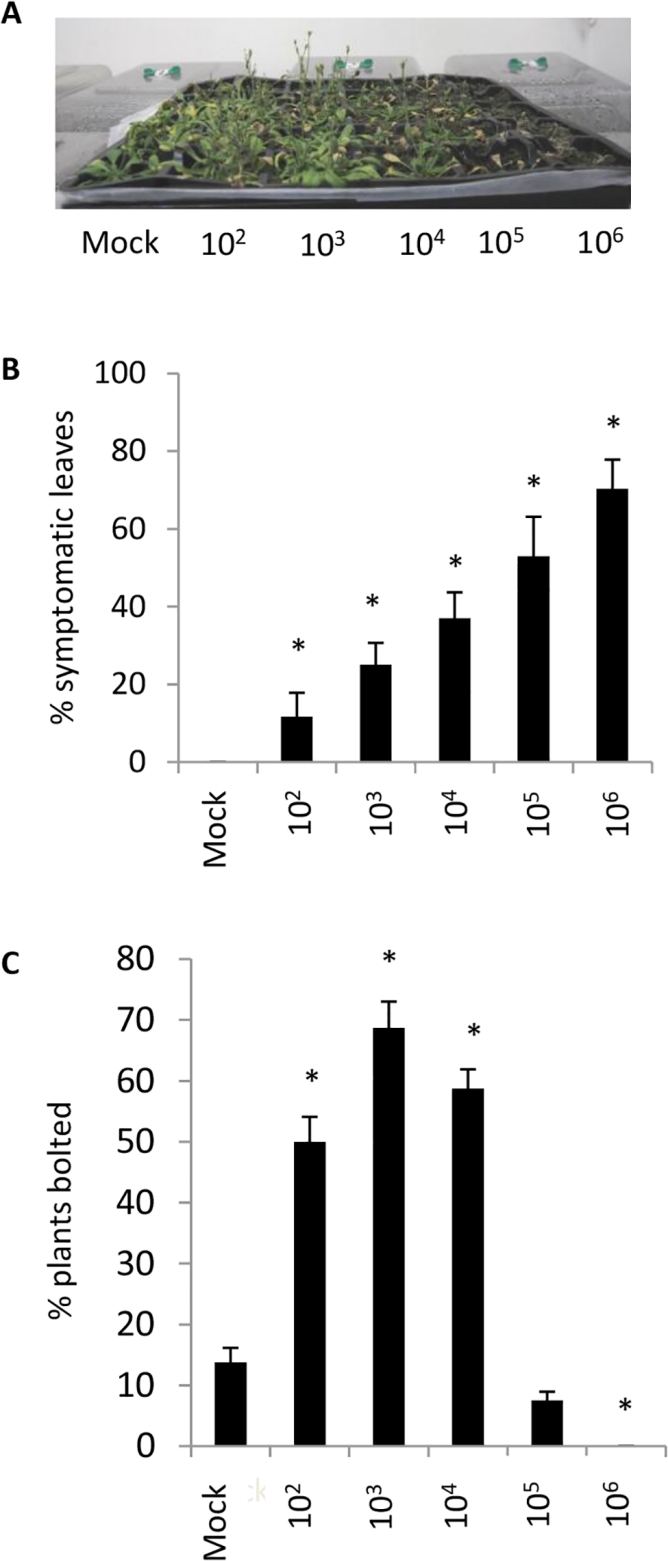
Moderate pathogen stress accelerates reproductive development in Arabidopsis. (A) Col-0 plants were inoculated with varying inoculum levels (Mock-10^6^ spores/mL) of the fungal pathogen *Fusarium oxysporum* at the 6–8 leaf rosette stage and photographed 14 days later (14dpi). (B) Percentage of leaves showing symptoms at 14 dpi. (C) Percentage of plants that had bolted at 14dpi in each treatment. Data from B-C show mean and standard error from three biological replicates each containing 10 plants per treatment. Asterisk indicates statistically significant difference from mock treatment (*P*<0.05) using a Student’s *t*-test.

To determine whether *F*. *oxysporum* affects the timing of the transition to flowering in *A*. *thaliana*, we infected Col-0 plants with serial dilutions of *F*. *oxysporum* spores and recorded disease severity and the proportion of plants that had bolted two weeks later. As expected, disease severity and plant death increased with the density of inoculum (Fig 1A and 1B). Plants infected with low to moderate densities of inoculum (10^2 –^ 10^4^ spores ml^−1^) showed a marked increase in the number of plants that had undergone the transition to flowering relative to the mock control (Fig 1C). Although the bolting response of plants inoculated with high inoculum densities could not be assessed due to plant death, overall these data suggest that the host plant is reprogrammed to accelerate the transition to flowering upon *F*. *oxysporum* infection.

### Late flowering and a high latitude is associated with enhanced *Fusarium oxysporum* resistance in geographically diverse *A*. *thaliana* ecotypes

A large variation in flowering time is known to exist within *A*. *thaliana* [45]. However, to date, a detailed analysis comparing the response of geographically diverse *A*. *thaliana* ecotypes to *F*. *oxysporum* has not been reported. To determine if an association could be found between flowering time and disease resistance, we obtained 83 publically available *A*. *thaliana* ecotypes in addition to the *F*. *oxysporum* susceptible ecotype Ty-0 [23] and assessed their response to *F*. *oxysporum*. The response to *F*. *oxysporum* was measured as a disease score relative to Col-0, with ecotypes showing statistically significantly lower scores than Col-0 considered as resistant and ecotypes showing significantly greater scores than Col-0 considered as susceptible.

**Fig 2:**
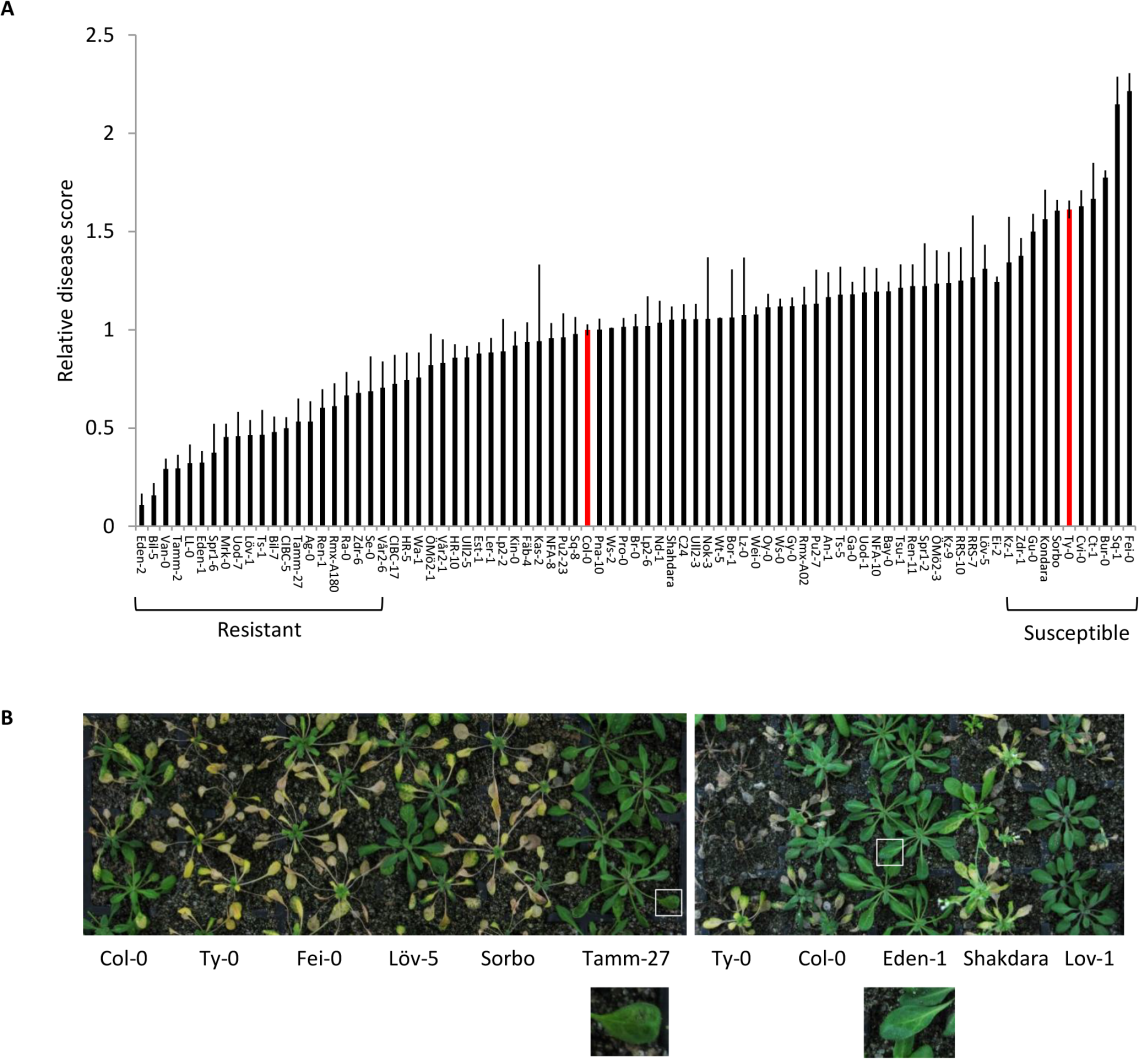
Response of *Arabidopsis* natural ecotypes to *F*. *oxysporum*. **(A)** *F*. *oxysporum* response was quantified by a disease score of 0–5 normalized to the disease score of the reference ecotype Col-0. Data shown are the mean and standard error of 3–40 plants per ecotype. Reference ecotype Col-0 and susceptible ecotype Ty-0 are shown in red. Ecotypes with disease scores significantly different (*P*<0.05) to that of Col-0 using a Student’s *t*-test were classed as resistant or susceptible. **(B)** Representative *F*. *oxysporum-* inoculated plants at 14 days post inoculation (dpi). Ty-0, Fei-0 and Sorbo are classed as susceptible, Col-0, Shakdara and Löv-5 are classed as intermediate and Tamm-27, Eden-1 and Löv-1 are classed as resistant. Enlarged photos of boxed leaves show vein clearing symptoms on highly resistant accessions Tamm-27 and Eden-1.

In total, 22 and 11 ecotypes were significantly more and less resistant than Col-0, respectively, while 50 ecotypes showed a similar disease score to Col-0 (Fig 2A). We found no evidence to suggest that complete immunity or incompatibility exists in the *F*. *oxysporum–A*. *thaliana* interaction since even the most resistant ecotypes displayed mild vein-clearing symptoms by 14 dpi (See Eden-1 and Tamm-27, Fig 2B).

**Fig 3:**
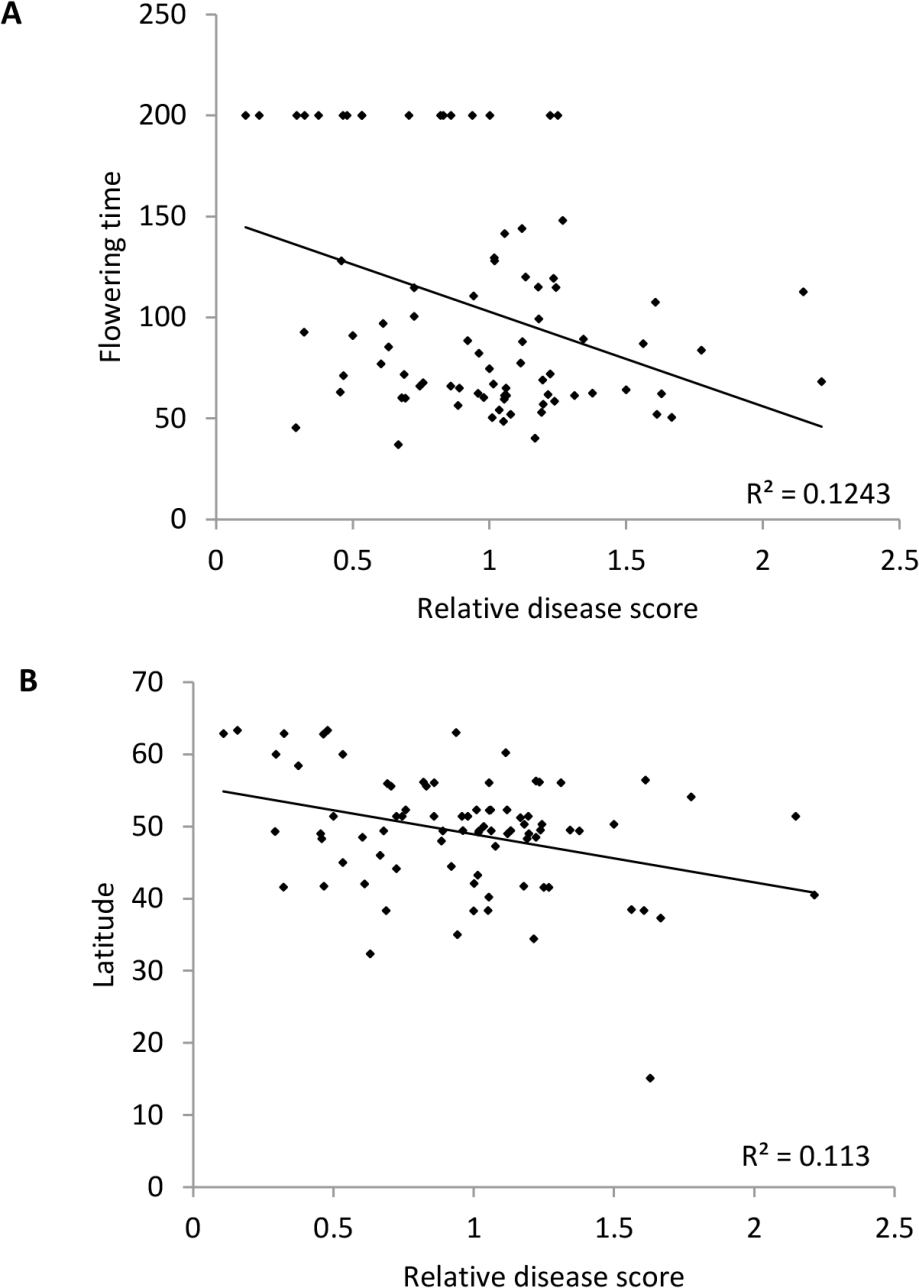
Correlation between flowering time or latitude and *Arabidopsis thaliana* accession response to *F. oxysporum*. (A) Flowering time was assessed as the number of days from germination until emergence of a 1cm bolt in ≥ 3 non-vernalized plants and was plotted against disease score for each of 83 natural accessions. The correlation using Pearson's product-moment correlation was significant (*P* = 0.003). (B) The disease score was plotted against latitude of the 83 natural accessions. The correlation using Pearson's product-moment correlation was significant (*P* = 0.005). Latitude information was obtained from https://easygwas.tuebingen.mpg.de/.

We recorded the flowering time (days to bolting) of each of the ecotypes and found a wide variation in flowering time, as previously reported [46]. Our flowering time data were generally consistent with publically available flowering-time phenotypes (http://www.arabidopsis.org/). To determine whether a relationship exists between flowering time and *F*. *oxysporum* resistance, we plotted flowering time against the *F*. *oxysporum* disease score for each of the *A*. *thaliana* ecotypes. This revealed a significant correlation between flowering time and *F*. *oxysporum* response such that late-flowering ecotypes showed enhanced resistance to *F*. *oxysporum* and the earlier flowering ecotypes were more susceptible (Fig 3A).

Given that the *A*. *thaliana* ecotypes used in this study are geographically diverse and have adapted to different environments, we looked for a relationship between the geographical origin and *F*. *oxysporum* response of the ecotypes by plotting the disease score of each of the ecotypes against latitude or longitude of origin. This analysis revealed that the disease score was not correlated with longitude (S1 Fig), however the disease score was significantly correlated with latitude (Fig 3B) such that enhanced disease resistance was associated with higher latitudes. Flowering time shows a latitudinal cline in *A*. *thaliana* natural ecotypes [46], and this was the case using our data (S2 Fig).

### Vernalization restores flowering time but not *F*. *oxysporum* response

**Fig 4:**
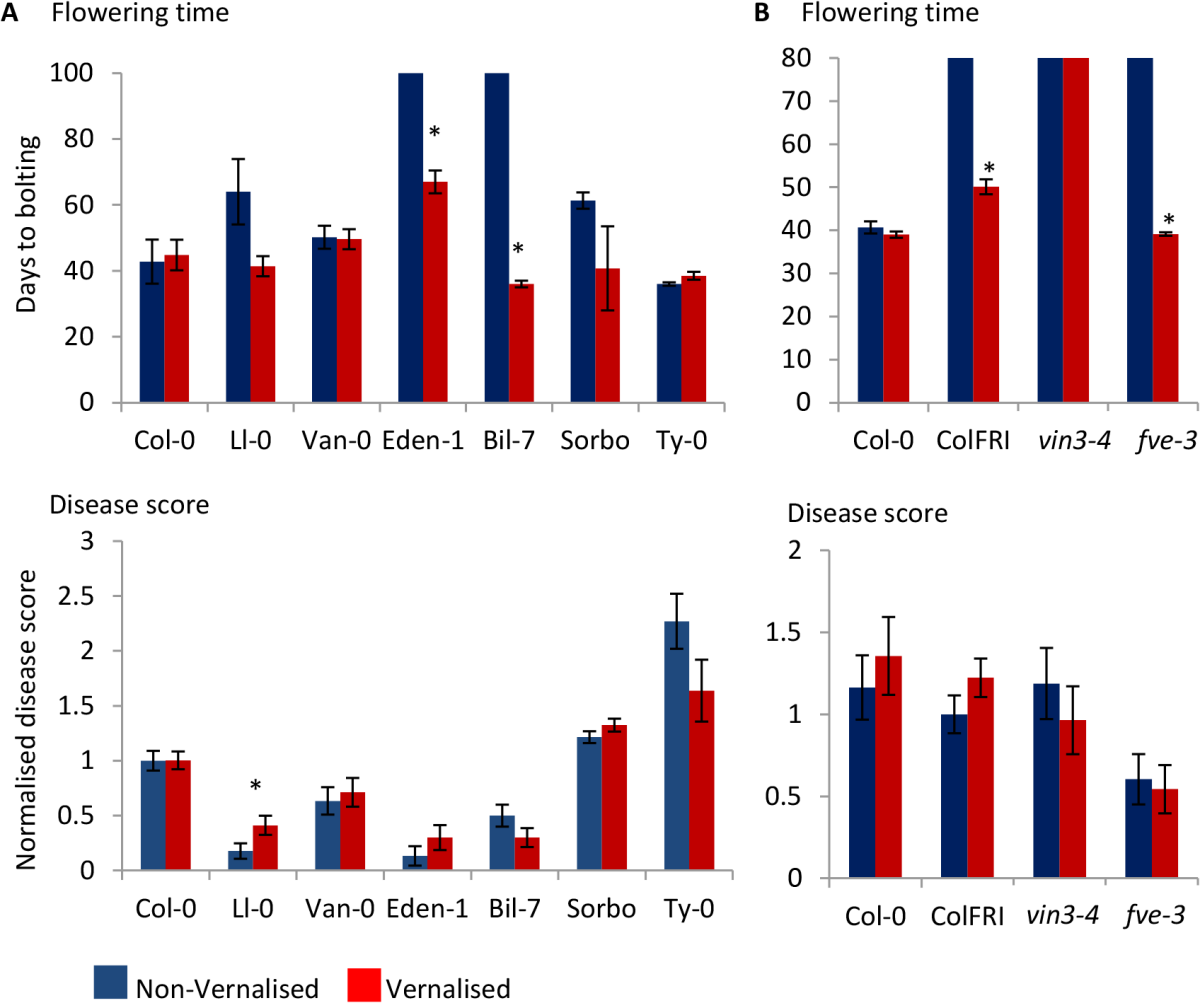
The role of vernalization in the *Arabidopsis thaliana* response to *F*. *oxysporum*. The effect of vernalization on flowering time and resistance to *F*. *oxysporum* in (A) *A*. *thaliana* natural accessions and (B) flowering-time and vernalization mutants. ‘Flowering time’ graphs display the number of days taken from germination to a 1 cm bolt. Data shown are mean and standard error from ≥3 plants per line. Plants that had not flowered at the conclusion of the experiment at 100 (A) or 80 (B) days post germination were given a value of 100 or 80. ‘Disease score’ graphs show mean and standard error of the disease score normalised to non-vernalized Col-0 (A) or non-vernalized ColFRI^SF2^ (ColFRI). (B). Asterisks indicate significant difference (*P*<0.05) between disease score of vernalized and non-vernalized plants using a Student’s *t*-test. Data shown are mean and standard error from >8 plants per line. Blue bars show data from non-vernalized plants; red bars show data from vernalized plants. The experiment was conducted twice and similar results were obtained each time.

We noted that vernalization-requiring Arabidopsis ecotypes derived from northern latitudes were highly represented among the resistant group ecotypes. Thirty-seven percent of resistant ecotypes were derived from Scandinavia as compared to 16% and 0% of ‘intermediate’ and susceptible ecotypes, respectively (Table A in S1 File). Since ecotypes adapted to these areas have a strong vernalization requirement, these findings indicated that inability to initiate flowering in the absence of vernalization may contribute to the resistance phenotypes observed in these ecotypes. We therefore asked whether accelerating flowering time by vernalization would render Arabidopsis more susceptible to *F*. *oxysporum*. We examined ecotypes Eden-1, Bil-7, Ll-0 that are both vernalization-sensitive and *F*. *oxysporum* resistant, ecotype Van-0 that is vernalization-insensitive and *F*. *oxysporum* resistant and Sorbo that is vernalization-sensitive and *F*. *oxysporum* susceptible. Vernalized plants of the Spanish ecotype Ll-0 flowered earlier (*P* = 0.06) and were more susceptible to *F*. *oxysporum* infection than non-vernalized plants, confirming the association between flowering time and *F*. *oxysporum* defense in this ecotype (Fig 4A). In contrast, although flowering time was accelerated in Swedish ecotypes Eden-1 and Bil-7, vernalized plants were as resistant as non-vernalized plants. Vernalization altered neither the flowering time nor response *to F*. *oxysporum* in the other ecotypes tested (Fig 4A). These data suggest that flowering time and *F*. *oxysporum* resistance phenotypes can be uncoupled in some natural Arabidopsis ecotypes.

Natural variation in flowering time and the vernalization requirement is largely mediated by allelic variation at FLOWERING LOCUS C (FLC) and FRIGIDA (FRI) [47]. *A*. *thaliana* ecotypes have generally evolved one of two life history strategies: ‘rapid cycling’ ecotypes, which can flower without vernalization and ‘winter’ ecotypes, which require vernalization to flower [48]. Most winter ecotypes contain functional FRI and FLC alleles, whereas many rapid cycling ecotypes have independently evolved null alleles at FRI or FLC [49–51]. ColFRI^SF2^ contains the FRI allele from the vernalization-sensitive *A*. *thaliana* ecotype San Feliu-2 introgressed into the Col-0 background, switching Col-0 from a rapid-cycling to a winter, vernalization-requiring ecotype [38]. To further explore the link between vernalization and disease resistance, we assessed the response of vernalized vs non-vernalized ColFRI^SF2^ plants to *F*. *oxysporum*. To account for potential crosstalk between cold exposure and defense, we included the *vin3-4* mutant unable to respond to vernalization in the ColFRI^SF2^ background [52]. As expected, vernalization accelerated flowering time in the late-flowering lines ColFRI^SF2^ but failed to alter flowering in the vernalization insensitive mutant *vin3-4*, which remained late flowering (Fig 4B). Similarly to the response of Eden-1 and Bil-7, subjecting ColFRI^SF2^ to vernalization prior to inoculations did not increase susceptibility to *F*. *oxysporum*. The vernalization-insensitive mutant *vin3-4* exhibited a WT (ColFRI^SF2^) response to *F*. *oxysporum* both before and after vernalization, suggesting that the prolonged cold exposure did not significantly influence disease progression in the ColFRI^SF2^ background. Furthermore, ColFRI^SF2^ plants were not more resistant to *F*. *oxsyporum* than Col-0 plants, suggesting that presence of a functional FRI does not contribute to resistance to *F*. *oxysporum*.

### Autonomous pathway mutants exhibit enhanced resistance to *F*. *oxysporum*

**Fig 5:**
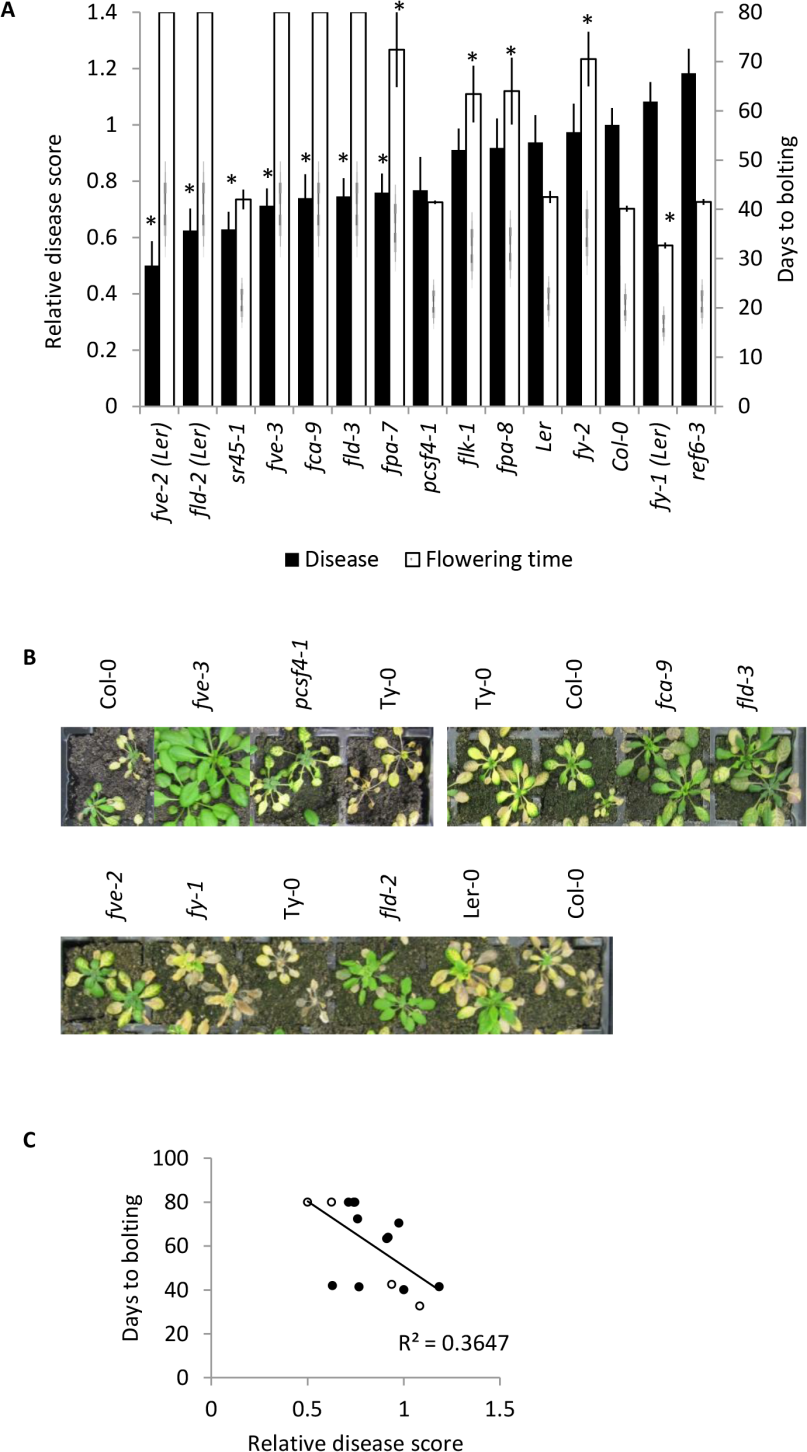
Response to *F*. *oxysporum* in autonomous pathway mutants. (A) *F*. *oxysporum* response (black bars) was quantified by a disease score of 0–5 normalised to the disease score of the reference ecotype Col-0. Disease score (black bars) show mean relative disease score at 14 days post inoculation (dpi) and standard error from at least 24 plants. Flowering time (white bars) was assessed as number of days taken from germination until emergence of a 1cm bolt. Plants that had not flowered at the conclusion of the experiment at 80 days post germination were given a value of 80. Data shown are mean and SE from ≥ 5 plants. Asterisks indicate significantly different values to WT (*P*<0.05) using a Student’s *t*-test. All mutants are in Col-0 background except those indicated (Ler). This experiment was conducted twice and similar results were obtained. (B) Representative *F*. *oxysporum* inoculated plants at 14 dpi. (C) Flowering time plotted against relative disease score for the 15 autonomous pathway mutant lines tested. Filled circles indicate Col-0 background, open dots indicate Ler background. The correlation using Pearson's product-moment correlation was significant (*P* = 0.0002). Experiments were conducted twice and similar results were obtained each time.

Members of the autonomous pathway promote flowering by down-regulating the floral repressor *FLC* independently of vernalization [53]. Thirteen loss-of-function mutants corresponding to nine members of the autonomous pathway were assessed for their response to *F*. *oxysporum* by comparing their disease score to the WT ecotype background. Under our growth conditions, *fve-2*, *fld-2*, *fve-3*, *flowering locus d-3*, *fca-9*, *flowering late kh motif-1 (flk-1)*, *fy-2*, *fpa-8* and *fpa-7* exhibited delayed flowering, whereas *relative of early flowering 6–3* (*ref6-3*), *pcf11p-similar protein 4–1* (*pcsf4-1)* and *serine arginine rich 45–1* (*sr45-1*) flowered at a similar time and *fy-1* showed an early flowering phenotype relative to their respective WTs (Col-0 or Ler-0) (Fig 5A). Seven mutants corresponding to five autonomous regulatory proteins: *fpa-7*, *fve-3*, *fve-2*, *sr45-1*, *fca-9*, *fld-2* and *fld-3* showed a resistant *F*. *oxysporum* phenotype relative to their respective WTs (Fig 5A and 5B). We plotted the relative disease score against the flowering time for each of the mutant and WT plants and found a positive correlation between late flowering via the autonomous pathway and a low disease score (Fig 5C). These data support the hypothesis that late flowering and *F*. *oxysporum* resistance are associated. Furthermore, given the absence of a latitude variable in this experiment, these data suggest that the association between flowering time and *F*. *oxysporum* response in natural ecotypes is unlikely to be caused by an indirect association between latitude and flowering time.

### A functional FLOWERING LOCUS C is not required for *F*. *oxysporum* resistance

**Fig 6:**
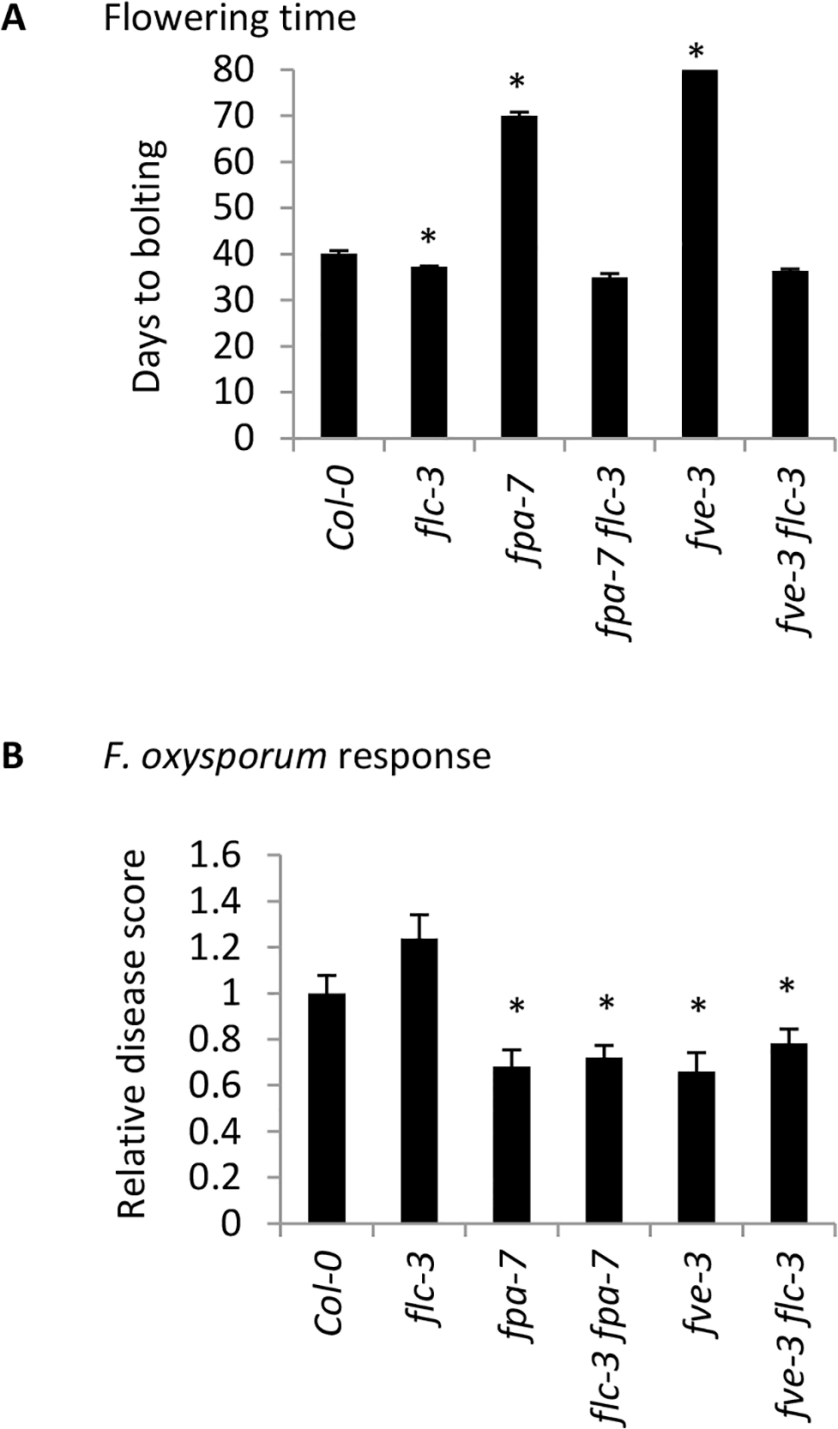
Role of FLC in the *F*. *oxysporum* response. (A) Flowering time data displayed are the number of days from germination to a 1cm bolt. Data shown are mean and standard error from ≥5 plants per line. Plants that had not flowered at the conclusion of the experiment at 80 days post germination were given a value of 80. Asterisk indicates significant difference to Col-0 (*P*<0.05) using a Student’s *t*-test. (B) *F*. *oxysporum* response was quantified by a disease score of 0–5 normalised to the disease score of the reference accession Col-0. Data show mean relative disease score at 14 days post inoculation and standard error from at least 24 plants. Asterisks indicate significantly different values to WT (*P* < 0.05) using a Student’s *t*-test. The experiment was conducted twice and similar results were obtained each time.

Given the importance of FLC in the mode of action of autonomous pathway genes, we specifically assessed the role of FLC in the *F*. *oxysporum* response using the FLC null mutant *flc-3*. The *flc-3* mutant flowered slightly but significantly earlier than Col-0 but showed a WT response to *F*. *oxysporum* infection (Fig 6). ColFRI^SF2^ exhibited a late-flowering phenotype relative to Col-0 under our conditions, but similarly to *flc-3*, showed WT response to *F*. *oxysporum* infection (Fig 4B).

We next investigated whether the late-flowering phenotype and elevated *FLC* levels of two autonomous pathway mutants *fve-3* and *fpa-7* are required for *F*. *oxysporum* resistance. The late-flowering phenotype of autonomous pathway mutants can be rescued by vernalization or loss of function of FLC. We compared the flowering time and disease response of single *fve-3* and *fpa-*7 mutants with *fve-3 flc-3* and *fpa-7 flc-3* double mutants. As expected, loss of *flc-3* restored the WT flowering time of *fve-3* and *fpa-7* mutants (Fig 6A). However, loss of *flc-3* in the *fve-3* or *fpa-7* background did not restore the WT susceptibility phenotype to *F*. *oxysporum*: double and single mutants were both more resistant to *F*. *oxysporum* than WT (Col-0) plants. Similarly, exposing *fve-3* plants to vernalization prior to infection accelerated flowering time but did not alter the response to *F*. *oxysporum* (Fig 4B).

These data, together with the vernalization experiments (Fig 4) suggest that the association between autonomously-controlled flowering time and *F*. *oxysporum* resistance can be uncoupled. They also indicate that neither FLC nor FRI regulate the response to *F*. *oxysporum* in the Col-0 background.

### FVE shows altered transcription of defense-related genes

The data presented above suggest that the effects of FPA and FVE on disease resistance or susceptibility most likely occur independently of FLC. Both FPA and FVE cause widespread epigenetic remodeling and transcriptional reprogramming in *A*. *thaliana* [34, 54, 55], so we reasoned that the enhanced *F*. *oxysporum* resistance phenotype seen in *fpa-7* and *fve-3* mutants could be pleiotrophic effect of processes other than flowering time. Indeed, FPA has previously been implicated in plant defense [17], but the role of FVE in plant defense has not been characterized. Given that *fve* mutants showed the strongest *F*. *oxysporum* resistance phenotypes of the autonomous mutants tested (Fig 5A and 5B), we investigated *fve-3* mediated resistance to *F*. *oxysporum* in more detail.

We identified genes differentially regulated >2 fold in *fve-3* plants relative to WT (Col-0) plants 6 days following inoculation either with water (mock treatment) or *F*. *oxysporum* (see complete list of differentially regulated genes (DEGs) in Table B in S1 File). *FVE* and *FLC* expression was down- and up-regulated, respectively, in *fve-3* in all tissues and treatments sampled, confirming the robustness of the experimental setup. Four hundred and eighty nine and 288 genes were differentially regulated in *fve-3* roots and shoots, respectively, after mock treatment, while 212 and 782 genes were differentially regulated in *fve-3* roots and shoots, respectively, after *F*. *oxysporum* treatment (Table 1). To understand the functionality of genes differentially regulated in *fve-3*, Gene Ontology (GO) term singular enrichment analysis was applied to DEGS in roots and shoots (Table C in S1 File). Among the genes that were differentially expressed in *fve-3* plants compared to Col-0 plants, genes involved in defense related functions were overrepresented. This was the case following either mock inoculation or *F*. *oxysporum* inoculation. The most highly overrepresented GO term among genes up-regulated in mock inoculated *fve-3* roots was ‘response to chitin’. Included in the chitin-responsive genes are ethylene response factors such as ERF2, which was previously implicated in *F*. *oxysporum* resistance [43] and several WRKY transcription factors that regulate defense [56, 57] (Table 2). The most highly overrepresented GO terms in genes up-regulated in mock inoculated *fve-3* leaves were related to phenylpropanoid and flavonoid biosynthesis and metabolism. Such compounds play important defensive roles in plants [58].

**Table 1:** Number of *fve-3* DEGs that are regulated by *F*. *oxysporum*.

Inoculation	Mock				*F*. *oxysporum*			
Tissue	Roots		Leaves		Roots		Leaves	
*fve-3*/Col-0	Up	Down	Up	Down	Up	Down	Up	Down
DEGS *fve-3/* Col-0 ^1^	306	183	127	160	133	79	569	215
Regulated by *F*. *oxysporum* ^2^	121	85	38	69	35	37	103	77
% *F*. *oxysporum* regulated	40	46	30	43	26	47	18	36

^1^ Differentially expressed genes (DEGs): genes that are >2 fold induced or repressed in fve-3 plants compared to Col-0 plants.

^2^ *fve-3* DEGs that are regulated >2 fold by *F*. *oxysporum* in Col-0 plants at 6 dpi (data deposited in SRA accession SRP052276).

**Table 2:** Defense associated genes up or down-regulated >2 fold in *fve-3* plants relative to WT plants.

		*fve-3* / Col-0			
		Mock		*F*. *oxysporum*	
Locus	Description	Roots	Leaf	Roots	Leaves
AT5G47220	ERF2	2.3	-	1.5	-
AT3G44260	CAF1a	3.0	0.3	2.1	1.4
AT2G39200	MLO12	-	-	-	2.3
AT3G45290	MLO3	-	-	-	2.0
AT2G30020	AP2C1	2.6	-	-	-
AT2G35980	NHL10 / YLS9	-	-	1.5	3.0
AT1G61340	FBS1	2.6	-	-	2.1
AT3G20600	NDR1	1.9	-	-	2.2
AT5G40990	GDSL LIPASE 1	-	-	2.2	2.0
AT5G43470	PPR8	-	-	5.0	-
AT4G12480	pEARLI 1	0.4	-	-	3.3
AT2G44490	PENETRATION 2	-	-	1.4	2.1
AT5G64905	PROPEP3	-	-	1.7	2.1
AT5G64890	PROPEP2	-	-	-	2.1
AT2G28830	PUB12	-	-	-	2.1
AT2G19190	FLG22-INDUCED RECEPTOR-LIKE KINASE 1	-	-	-	2.5
AT4G23190	CRK11	-	-	-	2.8
AT1G72300	LRR-RK	-	4.2	-	4.8
AT1G02450	NIMIN1	-	-	-	2.2
AT2G14610	PR1	-	-	-	3.6
AT2G19990	PR1-LIKE	2.6	-	-	-
AT1G75040	PR5	0.3	0.5	2.6	1.4
AT3G63360	DEFL-like	-	-	-	2.1
AT1G19610	PDF1.4	-	-	2.4	2.0
AT5G01900	WRKY62	7.4	-	1.7	-
AT5G22570	WRKY38	4.7	-	-	1.9
AT1G80840	WRKY40	2.0	0.5	1.6	1.8
AT4G23810	WRKY53	-	-	2.0	2.3
AT5G49520	WRKY48	1.5	-	-	3.2

Number indicates fold induction or repression in fve-3 roots or shoots relative to WT roots or shoots, respectively, 6 days after mock or *F*. *oxysporum* inoculation.

Several genes known to confer resistance in other plant-pathogen interactions were up-regulated in *fve-3* plants relative to Col-0 plants after *F*. *oxysporum* infection. Examples include GDSL LIPASE 1 (AT5G40990) that promotes ethylene-dependent resistance against fungal and bacterial pathogens [59], RECOGNITION OF PERONOSPORA PARASITICA 8 (AT5g42470) that promotes resistance against fungal and viral pathogens [60, 61], MLO12 and PEN2 that are required for resistance against powdery mildew [62] and NON RACE-SPECIFIC DISEASE RESISTANCE 1 that mediates resistance to *Pseudomonas syringae* [63]. Similarly, regulators of basal immunity including PROPEP2, PROPEP3, FRK1 and PUB12 were up-regulated in *fve-3* compared to Col-0 after *F*. *oxysporum* inoculation (Table 2).

pEARLI1 (AT4G12480) has antifungal properties against *F*. *oxysporum* [64] and was differentially expressed in *fve-3*. Interestingly pEARLI1 was previously shown to be differentially expressed in a number of late-flowering mutants from different floral promotion pathways [65]. AT1G72300 which encodes a leucine rich repeat receptor kinase involved in the perception of PSY1 that promotes susceptibility to *F*. *oxysporum* [66] was up-regulated >4-fold in leaves of mock and *F*. *oxysporum*-treated *fve-3* relative to Col-0 plants (Table 2).

Receptor-like proteins (RLPS) encode cell surface receptors that include components of innate immunity [66–68]. Nineteen of the 57 RLPS present in the Arabidopsis genome were up-regulated in *fve-3* plants after *F*. *oxysporum* infection, while six putative resistance gene homologues were up-regulated in *fve-3* plants after *F*. *oxysporum* infection (Table D in S1 File).

### *F*. *oxysporum* triggered transcriptional reprogramming of flowering-time regulators

To understand mechanisms underlying *F*. *oxysporum*-triggered acceleration of flowering time, we next asked if *F*. *oxysporum* infection alters the expression of flowering-time genes in the host. To achieve this aim, we mined RNA-seq data available from *F*. *oxysporum*-infected plants (data available at NCBI SRA, accession no. SRP052276) and identified plant floral regulator genes differentially expressed by *F*. *oxysporum* (Table 3). *FLOWERING LOCUS T* (*FT*) encodes a component of the mobile signal florigen that travels from the leaf to the meristem to initiate flowering and was induced by *F*. *oxysporum* infection. FLC, which represses *FT*, was also induced by *F*. *oxysporum* infection suggesting that *F*. *oxysporum*-mediated *FT* induction occurs independently of FLC. Floral promoters and repressors were both up- and down-regulated by *F*. *oxysporum*, suggesting that floral transition reprogramming in response to *F*. *oxysporum* infection undergoes fine-tuning and is under complex genetic control. Stress conditions often affect flowering through modulation of the photoperiodic pathway [69] and many of the *F*. *oxysporum*-regulated flowering-time genes belong to the photoperiodic pathway. The majority of these genes are also associated with the circadian clock which has been implicated in plant immune function [70].

**Table 3:** Flowering-time genes that are responsive to *F*. *oxysporum* infection.

		F1/M1		F6/M6	
Locus	Description	Roots	Shoots	Roots	Shoots
AT1G68050	FLAVIN-BINDING, KELCH REPEAT, F BOX 1	-	0.05	-	-
AT5G24470	PSEUDO-RESPONSE REGULATOR 5 (PRR5)	0.6	0.1	-	-
AT2G21660	GRP7	-	0.2	-	1.7
AT2G40080	EARLY FLOWERING 4 (ELF4)	-	0.3	-	-
AT3G07650	CONSTANS-LIKE 9 (COL9)	0.7	0.4	-	-
AT1G22770	GIGANTEA	0.7	0.4	1.5	2.5
AT5G39860	BASIC HELIX-LOOP-HELIX PROTEIN 136 (BHLH136)	-	0.6	-	0.4
AT2G30140	UDP-GLUCOSYL TRANSFERASE 87A2 (UGT87A2)	-	1.6	2.2	2.0
AT2G39250	SCHNARCHZAPFEN (SNZ)	-	2.1	-	2.8
AT5G23730	EARLY FLOWERING BY OVEREXPRESSION 2 (EFO2)	-	2.1	-	-
AT5G10140	FLOWERING LOCUS C	2.2	2.7	0.6	-
AT2G46830	CIRCADIAN CLOCK ASSOCIATED 1 (CCA1)	1.7	3.1	0.6	-
AT1G65480	FLOWERING LOCUS T (FT)	-	3.8	-	-
AT5G01040	LACCASE 8 (LAC8)	-	-	2.4	-
AT2G24790	COL3	-	-	0.4	-
AT4G14900	FRIGIDA-like	-	-	0.2	-
AT1G09570	PHYA	-	-	-	0.4
AT1G71692	AGAMOUS-LIKE 12 (AGL12)	-	-	1.7	5.7
AT5G63980	HIGH EXPRESSION OF OSMOTICALLY RESPONSIVE GENES 2	1.4	-	-	2.7
AT5G24860	ARABIDOPSIS FLOWERING PROMOTING FACTOR 1 (ATFPF1)	2.3	-	-	-

Number indicates fold induction or repression by *F*. *oxysporum* relative to mock treatment in Col-0 tissue at 1 (F1/M1) or 6 days (F6/M6) after inoculation.

### Flowering-time regulator GI acts as a susceptibility factor for *F*. *oxysporum* infection

**Fig 7:**
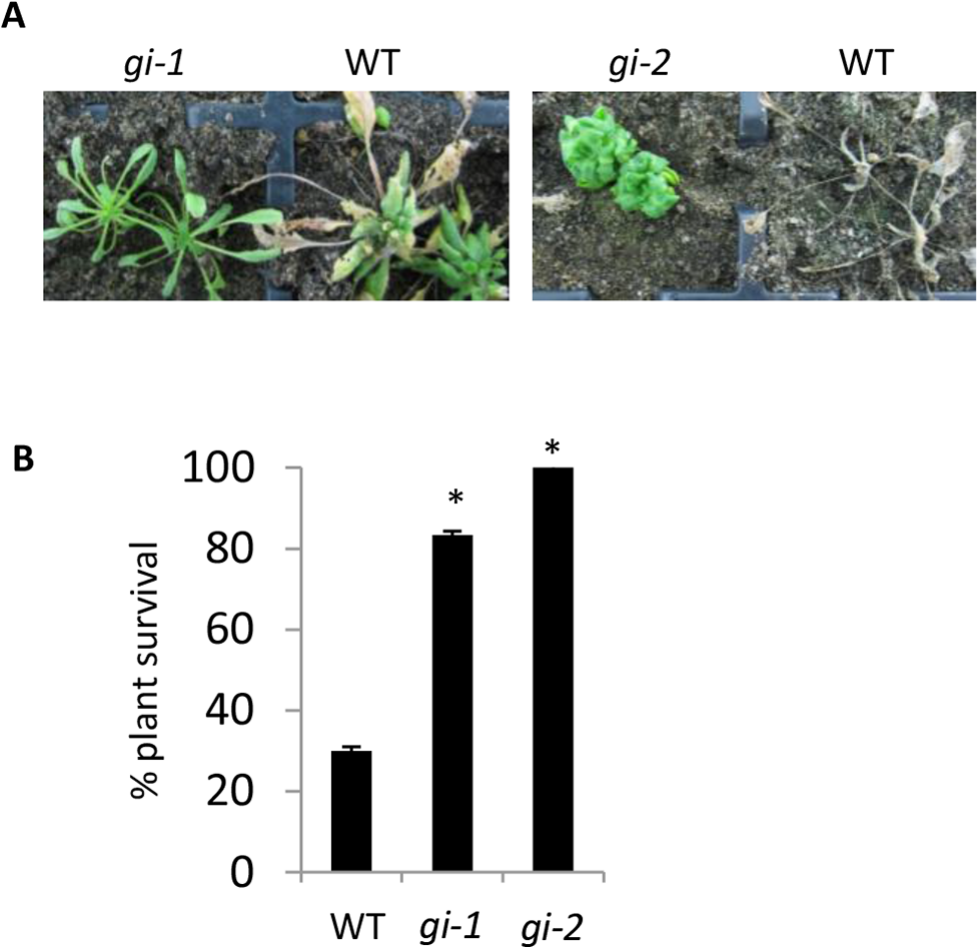
GIGANTEA promotes susceptibility to *F*. *oxysporum*. (A) Representative *F*. *oxysporum-* inoculated WT and mutant plants at 14 days post inoculation (dpi). (B) Percentage plant survival at 21 dpi. Data shown are mean and standard error from 3 biological replicates each containing 10 plants per line. Asterisks indicate significant difference relative to WT (*P*<0.05) using a Student’s *t*- test.

One of the photoperiodic pathway flowering-time regulators responsive to *F*. *oxysporum* was GIGANTEA (GI) and this factor promotes flowering by directly activating *FT* [71]. To determine if GI affects disease resistance, we inoculated two independent *gi* mutants with *F*. *oxysporum* and scored disease development. Both *gi* mutants showed increased resistance to *F*. *oxysporum* (Fig 7), suggesting that GI acts as a susceptibility factor in this interaction.

## Discussion

**Fig 8:**
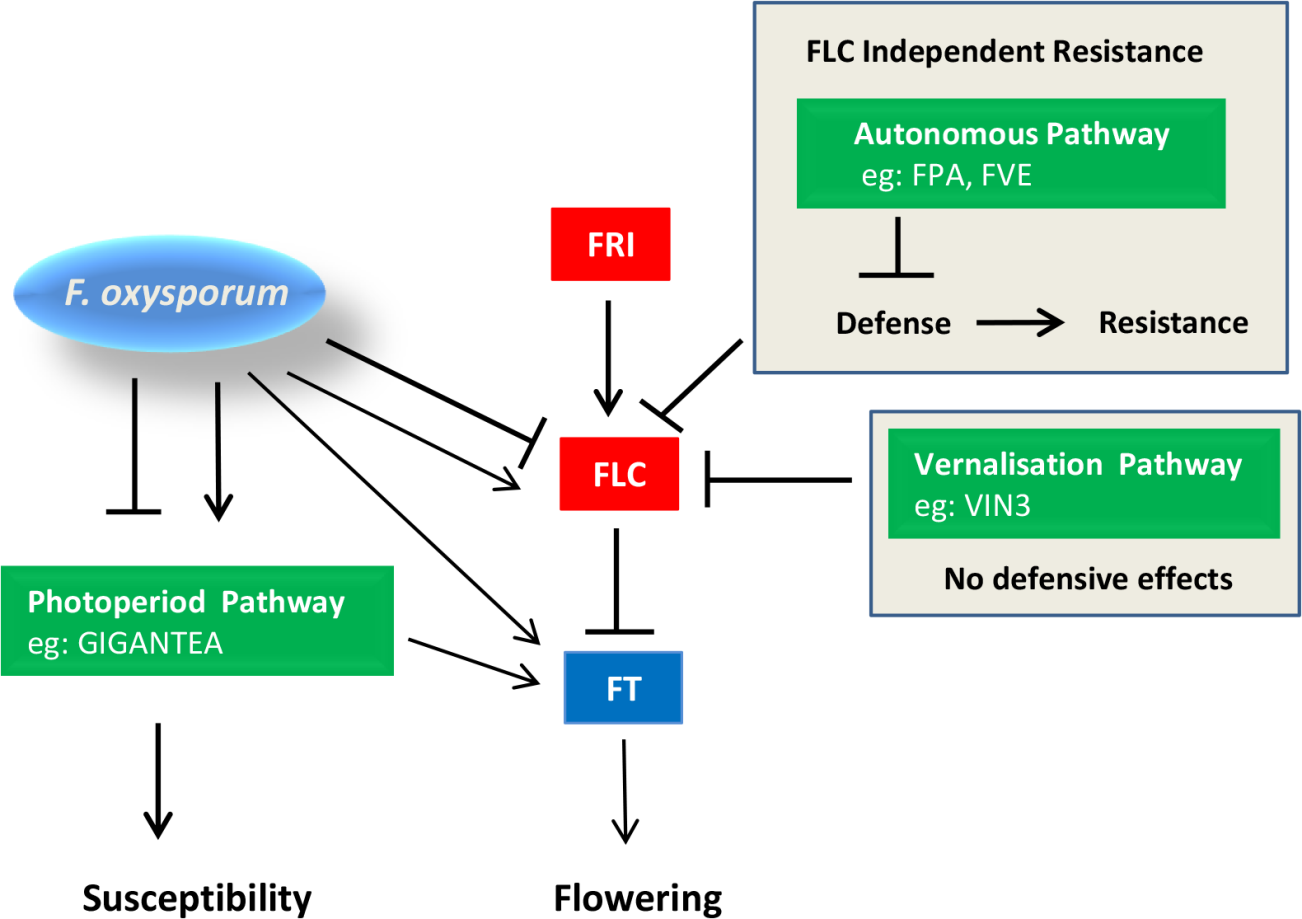
Crosstalk between flowering-time regulators and *F*. *oxysporum* response in *Arabidopsis thaliana*. Diagram represents a highly simplified schematic of the transition to flowering in *Arabidopsis thaliana* and the transcriptional affect of *F*. *oxysporum* on flowering-time genes relevant to this study. During long days, the mobile signalling component FT travels from the leaf to the meristem to initiate flowering. The FLOWERING LOCUS C (FLC) antagonizes the flowering transition by repressing *FT* and other floral integrators. In winter annuals, *FLC* transcription is activated by the plant-specific protein FRIGIDA (FRI). *FLC* transcription is down-regulated by vernalization (exposure to prolonged cold) or by members of the autonomous pathway at ambient temperatures, allowing flowering to occur under conducive conditions.

Increasingly, studies are reporting cross-talk between stress response and the transition to flowering [72–74], but mechanisms underlying stress-induced flowering changes or modulation of stress responses by flowering-time integrators are poorly understood. The objective of this study was to investigate the relationship between defense and flowering time using the *F*. *oxysporum* – Arabidopsis interaction. The key findings of this study are summarized in the working model proposed in Fig 8.

*F*. *oxysporum* inoculation causes transcriptional reprogramming of flowering-time genes, particularly those in the photoperiod pathway, with the net effect of accelerating flowering time. Arrows from *F*. *oxysporum* to flowering-time genes indicate transcriptional regulation as shown in Table 3. Autonomous pathway floral regulators that regulate flowering time by repressing *FLC*, are not transcriptionally responsive to *F*. *oxysporum* infection, but several members of this pathway promote susceptibility to *F*. *oxysporum*. *fve-3* shows altered defense gene transcription, suggesting that FVE and potentially other autonomous pathway mutants are compromised in defense against *F*. *oxysporum*. GIGANTEA (GI), which promotes flowering time independently of FLC, promotes susceptibility to *F*. *oxysporum*.

*FLC* is responsive to *F*. *oxysporum* infection, however FLC *per se* does not seem to modulate the *F*. *oxysporum* response. FRI and members of the vernalization pathway are not transcriptionally regulated by *F*. *oxysporum* and do not appear to regulate the *A*. *thaliana* response to *F*. *oxysporum*.

We discovered a negative association between flowering time and resistance to *F*. *oxysporum* using natural ecotypes and mutant lines of *A*. *thaliana* and hypothesized that the correlation between delayed flowering and *F*. *oxysporum* resistance could be due to a pleiotrophic effect of delayed senescence in late-flowering lines, minimizing the disease symptoms caused during the necrotrophic phase of infection. Indeed an association between enhanced senescence and disease susceptibility has been reported in the *F*. *oxysporum—A*. *thaliana* interaction [75, 76]. An overabundance of vernalization-requiring ecotypes were resistant to *F*. *oxysporum*, so we investigated the role of vernalization, and vernalization-associated genes FRI and FLC, in the response to *F*. *oxysporum*. When flowering time was accelerated in late-flowering lines by FLC knockout or vernalization, or delayed by the addition of FRI in Col-0, the *F*. *oxysporum* response phenotype was unchanged, suggesting that neither the late-flowering phenotype nor FLC or FRI are required for resistance to *F*. *oxysporum*. These findings challenge the hypothesis that *F*. *oxysporum* resistance in late-flowering lines is a pleiotrophic effect of delayed senescence. Rather, they suggest that genes controlling flowering time may have dual functionality in defense regulation via genetically distinct pathways.

The *fpa-7* and *fve-3* mutants exhibit *F*. *oxysporum* resistance independently of FLC. FPA is an RNA binding protein and loss of function of FPA results in genome-wide RNA processing changes [34, 77]. FVE is a WD40 scaffold protein that is a component of several nucleoprotein complexes that mediate epigenetic modifications on a genome-wide scale [78–80]. FVE and FPA therefore play general roles in transcriptional regulation and are likely to have a broad target range which includes FLC as well as other genes. We reasoned that the *F*. *oxysporum* resistance phenotype observed in *fve-3* could be a pleiotrophic effect of a process other than flowering modulated by FVE. FVE is known to regulate the cold acclimation response [81] and several cold responsive genes were recently shown to be transcriptionally regulated by *F*. *oxysporum* in Col-0 plants (data available in SRA accession SRP052276). However, cross-talk between the cold acclimation pathway and *F*. *oxysporum* response is not well understood and requires further investigation. Using RNA-seq, we were able to ascertain that *fve-3* mutants show up-regulation of chitin responsive and glucosinolate-associated genes (Table C in S1 File) even in the absence of pathogen stress, suggesting that these plants are primed to respond more quickly to fungal attackers. Six days after inoculation with *F*. *oxysporum*, *fve-3* plants also exhibited up-regulation of key defense regulators relative to Col-0 plants (Table 2), suggesting a higher amplitude of the defense response.

Similarly to our findings that FLC and late flowering can be uncoupled from resistance in autonomous pathway mutants *fpa*-7 and *fve-3*, it was recently shown that *enhanced downy mildew2* (*edm2*) and *fld* mutants require FLC for late flowering but not disease resistance phenotypes [18, 82]. The authors proposed that EDM may have evolved to perform functions in addition to its role in flowering-time regulation [82] and such a scenario is also plausible in the case of FVE or FPA. Increasingly in the literature, flowering-time mutants are being shown to have vegetative phenotypes related to defense [17, 18, 65, 82]. Indeed, FLC itself targets hundreds of genes unrelated to flowering such as JAZ proteins, which mediate JA signaling [83].

In addition to FLC and FRI, members of the photoperiod pathway also contribute to natural variation in flowering time in *A*. *thaliana* [84–87]. We found that a number of flowering regulators from the photoperiod pathway respond to *F*. *oxysporum* infection. Of these, GI, together with CO and FT promotes flowering in a circadian-clock controlled manner [88]. Our mutant analyses indicate that GI promotes susceptibility to *F*. *oxysporum*. Further research is required to determine whether *gi*-mediated resistance is a due to delayed flowering or a result of a pleotrophic effect of other processes regulated by GI such as cytokinin signaling [89], sucrose signaling [90], oxidative stress response [91], salinity tolerance [92], freezing tolerance [93], drought escape response [74] or response to viral pathogens [94].

Flowering time is a highly complex trait mediated by multiple genetic pathways. This study focused on the role of the photoperiodic and FLC-dependent flowering-time pathways in the *A*. *thaliana—F*. *oxysporum* interaction but was not an exhaustive examination of flowering-time regulators. Members of the GA flowering pathway are known to regulate JA/SA signaling [21] while SVP, a member of the thermosensory flowering pathway, modulates age-related resistance to *P*. *syringae* [95] and the roles of these genes in the response to *F*. *oxysporum* were not tested here.

Providing adequate disease protection in order to maintain reproductive success is paramount to achieving agricultural productivity. We have demonstrated that inoculation with a moderate concentration of *F*. *oxysporum*, which may represent the situation in nature, can accelerate flowering time in Arabidopsis. Evidence suggests that global warming has already affected the flowering time of many plant species [96–98]. Simulated future seasonal warming accelerated flowering and even prompted switching of life history strategies from ‘winter’ to ‘rapid cycling’ in *A*. *thaliana* natural ecotypes [99]. Climate change is also predicted to alter the severity of plant disease epidemics [100]. An increased knowledge of genetic mechanisms underlying the interaction between flowering time and defense in crop plants will assist breeders to manage these two traits to accomplish the best agricultural outcomes in the future.

## Supporting Information

S1 Fig Correlation between longitude and disease score in *Arabidopsis thaliana* natural ecotypes. The disease score was plotted against longitude of the 97 natural accessions. The correlation using Pearson's product-moment correlation was not significant (*P* = 0.08). Longitude information was obtained from https://easygwas.tuebingen.mpg.de/.
(PDF)

S2 Fig Correlation between flowering time and latitude in *Arabidopsis thaliana* natural ecotype. Flowering time was assessed as number of days taken from germination until emergence of a 1cm bolt in ≥ 2 non-vernalized plants and was plotted against latitude each of 97 natural accessions. The correlation using Pearson's product-moment correlation was significant (*P* = 2.844e-05).
(PDF)

S1 File Table A. Number of *F*. *oxysporum*-resistant, intermediate and susceptible genotypes originating from Scandinavia. Table B. Genes that are differentially regulated >2 fold in *fve-3* plants relative to Col-0 plants after mock and/or *F*. *oxysporum* treatment. Table C. Results of gene ontology (GO) singular enrichment analysis (SEA) showing the five most significantly overrepresented functional gene ontology categories in *fve-3* relative to WT (Col-0) plants in roots or shoots after mock or *F*. *oxysporum* inoculation. Table D. Receptor like proteins (RLPs) and NBS-LRR genes differentially expressed in *fve-3* plants relative to WT plants. Number indicates fold induction or repression in *fve-3* roots or shoots relative to WT roots or shoots, respectively, 6 days after mock or *F*. *oxysporum* inoculation.
(XLSX)
