# Supplementary figures and images for: Investigating the Association between Flowering Time and Defense in the Arabidopsis thaliana-Fusarium oxysporum Interaction
Source: PLoS One. 2015 Jun 2;10(6):e0127699. doi: 10.1371/journal.pone.0127699 (PMC4452756; doi:10.1371/journal.pone.0127699)

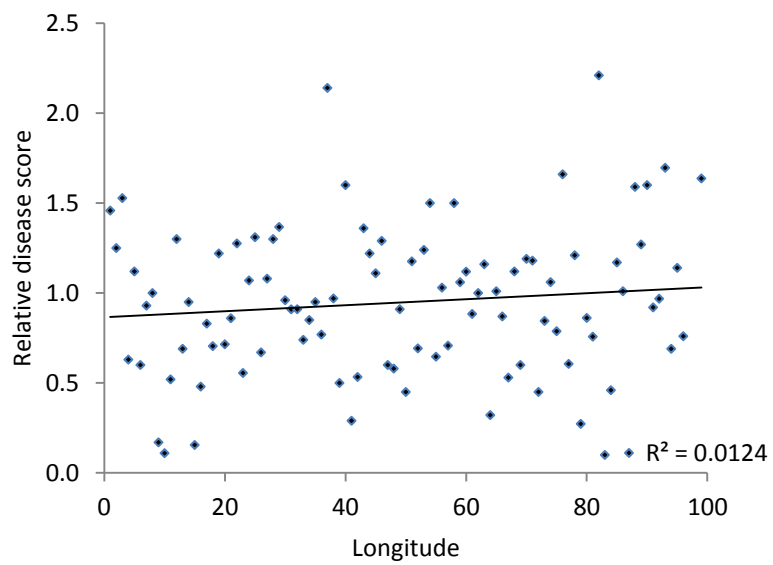

Supplement: S1 Fig — The disease score was plotted against longitude of the 97 natural accessions. The correlation using Pearson's product-moment correlation was not significant (P = 0.08). Longitude information was obtained from https://easygwas.tuebingen.mpg.de/. (PDF) [file pone.0127699.s001.pdf]

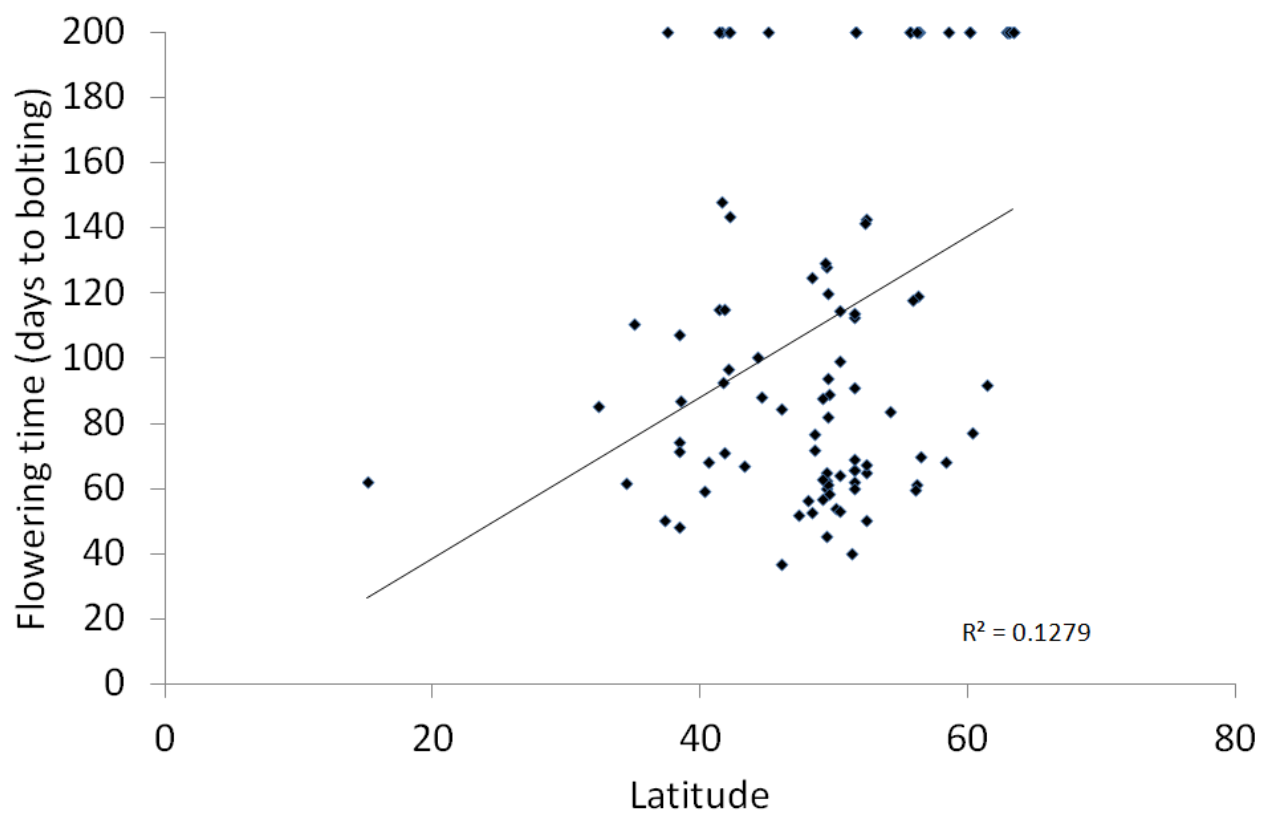

Supplement: S2 Fig — Flowering time was assessed as number of days taken from germination until emergence of a 1cm bolt in ≥ 2 non-vernalized plants and was plotted against latitude each of 97 natural accessions. The correlation using Pearson's product-moment correlation was significant (P = 2.844e-05). (PDF) [file pone.0127699.s002.pdf]
